# Expression Pattern Analysis of Antiviral Genes and Inflammatory Cytokines in PEDV-Infected Porcine Intestinal Epithelial Cells

**DOI:** 10.3389/fvets.2020.00075

**Published:** 2020-02-18

**Authors:** Shiqin Wang, Jiayun Wu, Fang Wang, Haifei Wang, Zhengchang Wu, Shenglong Wu, Wenbin Bao

**Affiliations:** ^1^Key Laboratory for Animal Genetics, Breeding, Reproduction and Molecular Design of Jiangsu Province, College of Animal Science and Technology, Yangzhou University, Yangzhou, China; ^2^Joint International Research Laboratory of Agriculture & Agri-Product Safety, Yangzhou University, Yangzhou, China

**Keywords:** pigs, PEDV, innate immune response, antiviral genes, inflammatory cytokines

## Abstract

Porcine diarrhea disease in newborn and suckling piglets due to infection with porcine epidemic diarrhea virus (PEDV) is a leading cause of economic loss in the pig industry globally. In this study, we investigated the molecular mechanism of the host innate immune response to PEDV infection. The expression dynamics of antiviral genes (e.g., *RIG-1, PKR, OAS1, Mx1*, and *Mx2*) and inflammatory cytokines (e.g., *IFN-*α*, IFN-*β, *TNF-*α, *IL-6, IL-8*, and *IL-12*) in porcine small intestinal epithelial (IPEC-J2) cells were analyzed following PEDV stimulation. The results showed that the expression of antiviral genes (e.g., *PKR, OAS1*, and *Mx2*) and inflammatory cytokines (e.g., *IFN-*α and *TNF-*α) were significantly reduced within 0–4 h post-infection (*P* < 0.05). However, all antiviral genes and inflammatory cytokines were up-regulated from 12 to 24 h (*P* < 0.05), and cytopathic changes were observed during this time. The expression of *RIG-1, PKR, OAS1, Mx1*, and *Mx2* were significantly and positively correlated to each other during the entire infection (*P* < 0.01). The results suggested that the *RIG-1, PKR, OAS1, Mx1*, and *Mx2* genes may play an important role in PEDV infection in piglets. Initially, PEDV displayed cellular invasion by inhibiting *IFN-*α transcription and interfering with the antiviral function of *PKR, OAS1*, and *Mx2*, ultimately induced an intense inflammatory response. The relationship between antiviral genes and inflammatory cytokines with PEDV infection at the cellular level provides a reference for studying the mechanism of resistance to PEDV infection in piglets.

## Introduction

Porcine epidemic diarrhea (PED) is a swine infectious disease characterized by levels of susceptibility, high incidence, and fatality. PED was first discovered in the United Kingdom in 1971 and successively erupted in Europe, Asia, America, and other regions within the next 40 years (1–5). Moreover, PED has become an important epidemic that restricts the development of the pig industry, causing serious economic losses. Porcine epidemic diarrhea virus (PEDV) is the causative agent of PED, which was discovered and termed, CV777, in 1978 (6). PEDV invades the digestive system of pigs through the nose and mouth, targeting, and residing in the porcine small intestinal epithelial cells. PEDV subsequently destroys the mucosal immune barrier of the small intestine, finally causing watery diarrhea, dehydration, and vomiting in piglets. The morbidity and mortality of newborn and suckling piglets reach 100% (7). Currently, the control measures for PED mainly include the vaccination of pregnant sows; however, neither attenuated nor inactivated vaccines have been significantly effective in practice (8, 9).

The innate immune response is the first line of host defense against pathogen invasion. PEDV is a coronavirus, a single-stranded positive-sense RNA virus, which is recognized and activates by retinoic acid-inducible gene I (RIG-I) -like receptors I. RIG-I activation induces the production of type I interferon (IFN-I) to regulate the expression of a series of interferon stimulated genes (ISGs) that stimulate target cells to produce specific antiviral proteins (e.g., double-stranded RNA-dependent protein kinase [PKR], 2-5′ oligoadenylate synthase 1 [OAS1], and myxovirus resistance proteins [Mx]) (10–12). Proinflammatory cytokines (e.g., tumor necrosis factor [TNF-α] and interleukin 6 [IL-6]) are also produced to mediate various inflammatory responses and exert antiviral effects (13). Therefore, further exploration of the host innate immune response following PEDV infection can provide a theoretical basis for the study of piglet resistance to PEDV infection.

In the present study, porcine small intestinal epithelial cells were used as an *in vitro* model to investigate PEDV infection. Real-time quantitative polymerase chain reaction was used to detect differences in the expression of the *RIG-1, PKR, OAS1, Mx1*, and *Mx2* genes at various time points post-infection. In addition, the mRNA levels of some inflammatory factors was also determined. The purpose of this study was to investigate the molecular regulation related to the host innate immune system that are mediated by PEDV infection, and provide a theoretical basis for studying the mechanism of resistance to PEDV infection in piglets.

## Materials and Methods

### Experimental Materials

The porcine small intestinal epithelial cell line, IPEC-J2, was kindly provided by the University of Pennsylvania, USA. Vero cells were purchased from the National Center for Type Culture Collection. The PEDV CV777 strain was kindly provided by the China Agriculture University. Complete DMEM medium containing 10% fetal bovine serum FBS, trypsin-EDTA solution, and cell culture well-plates were purchased from Gibco (USA). Dry powder PBS phosphate buffer was purchased from Beijing Saintan Technology Co., Ltd. (Beijing, China). TRIzol reagent was purchased from Invitrogen (USA). Reverse transcription and quantitative fluorescence kits were both purchased from Vazyme Biotech Co., Ltd. (Nanjing, China).

### Primer Design and Synthesis

According to the gene sequences published in the GenBank database of antiviral genes *RIG-1, PKR, OAS1, Mx1*, and *Mx2* and inflammatory factors *IFN-*α, *IFN-*β, *TNF-*α, *IL-6, IL-8*, and *IL-12* genes, Primer Premier 5.0 software was used to design real-time PCR primers across gene exons. The *GAPDH* gene was used as an internal control. Primer information was listed in Table S1. The primers were all synthesized by Sangon Biological Engineering Co., Ltd. (Shanghai, China).

### Expansion and Collection of PEDV

Vero cells were inoculated onto a 6-well plate at a density of 2.5 × 105 cells per well, and cultured in DMEM complete medium containing 10% FBS at 37°C in a CO_2_ incubator until the density reached 90%. The PEDV CV777 strain was inoculated into Vero cells and cultured in a 500-μL viral solution (MOI = 0.1) per well at 37°C and 5% CO_2_ for 2 h. Next, the supernatant was absorbed and 2 mL DMEM complete medium was supplemented into each well for 72 h. Finally, the virus was collected after observing the obvious pathological changes of the infected Vero cells under a microscope. The 6-well plate was frozen in the refrigerator at −20°C and then melted at room temperature on a clean bench. After three rounds of freezing and thawing, the Vero cells were lysed to release the virus. The harvested virus suspension was collected into a 15 mL centrifuge tube. The cells were centrifuged at 2,000 r/min for 5 min to remove cell debris, and the supernatant was divided into a 1.5-mL centrifuge tube and stored at −80°C.

### PEDV Infection of IPEC-J2 Cells

IPEC-J2 cells were inoculated into a 12-well plate at a density of 1 × 10^5^ cells per well, and cultured in complete DMEM medium containing 10% FBS in a CO_2_ incubator until they reached ~90% confluence. At the same time, the cells in the control group were collected for subsequent total RNA extraction. The culture medium was discarded, the cells were washed twice in PBS, and 300 μL of the PEDV solution (MOI = 0.1) was added into each well. Three replicates were placed in each group and cultured for 2 h. The culture medium was replaced with 500 μL of fresh complete medium and cultured for 2, 10, or 22 h, respectively. The pathological changes of the cells during each infection period were observed under a microscope.

### Total RNA Extraction and cDNA Synthesis

Using the TRIzol method, the total RNA was extracted in strict accordance with the manufacturer's protocol. RNA degradation and contamination was monitored on 1% agarose electrophoresis and NanoDrop ND-1000 nucleic acid/protein concentration meter (GE, USA), followed by storage at −80°C until further use.

According to the instructions in the reverse transcription kit, the reaction system consisted of synthesizing cDNA in 20 μL reactions containing 4 μL qRT SuperMix II (5×) and 1,000 ng total RNA, with the remaining volume comprised of RNase-free ddH_2_O. The cycling parameters were as follows: 25°C for 10 min, 50°C for 30 min, and 85°C for 5 min. The reaction mixtures were stored at 4°C.

### Real-Time PCR Reaction

The 20 μL reaction system included 10 μL SYBR Premix ExTapTM II (2×), 0.4 μL for upstream and downstream primers (10 μmol/L), 0.4 μL ROX Reference Dye II (50×), 2 μL cDNA, and 6.8 μL RNase-free ddH_2_O with a thermocycling program of 40 cycles of 95°C for 30 s, 95°C for 5 s, and 60°C for 34 s. To analyze the specificity of the amplified products, a melting curve analysis was executed after amplification as follows: 95°C for 15 s, 60°C for 1 min; 95°C for 15 s, and 60°C for 15 s.

### Prediction of Protein-Protein Interaction Networks

Using the interaction network database, String 11.0 (https://string-db.org/), the interaction between porcine RIG-1 (also known as DDX58), PKR (also known as EIF2AK2), OAS1, Mx1, and Mx2 proteins was analyzed.

### Data Statistics and Analysis

The relative quantitative results were analyzed and processed via the 2^−ΔΔ*Ct*^ method (14). The level of internal reference gene, GAPDH, expression was used to homogenize the target gene. The univariate statistical analysis method of the SPSS 18.0 software general linear model was used to compare and analyze the expression levels of the *RIG-1, PKR, OAS1, Mx1*, and *Mx2* genes and the transcription levels of *IFN-*α, *IFN-*β, *TNF-*α, *IL-6, IL-8*, and *IL-12* in IPEC-J2 cells at various time points post-infection. A correlation analysis was performed pairwise for all relevant genes using a Pearson correlation, *P* < 0.05 was deemed significant.

## Results

### Determination of the Total RNA Integrity and Purity

The level of RNA purity was tested by 1% agarose electrophoresis. The results indicated that the extracted RNA was of high quality without obvious degradation or DNA contamination (Figure S1). In addition, the OD260/OD280 value detected by NanoDrop ND-1000 was between 1.8 and 2.0 and the concentration was between 100 and 300 μg/μL. Overall, these results demonstrated that the extracted RNA was suitable for further analysis.

### Amplification and Melting Curves for Real-Time PCR

According to Figures S2A,B, the PCR products had only one specific peak, without primer dimers and non-specific products, which indicated that all of the target genes had been successfully amplified.

### Pathological Changes in IPEC-J2 Cells After PEDV Infection

**Figure 1:**
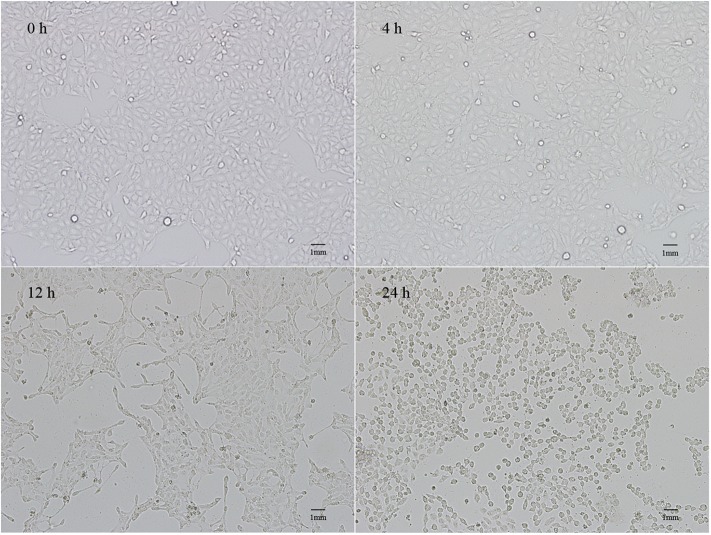
Lesions of IPEC-J2 cells infected with PEDV for 4, 12, and 24 h.

The pathological changes in PEDV-infected cells were observed under a microscope (Figure 1). Normal IPEC-J2 cells were full fusiform with clear boundaries and no overlap. No obvious changes were observed at 4 h after PEDV infection. However, 12 h later, cell shrinkage, elongation, and fusion occurred, which presented as typical cell lesions. Infection for 24 h caused serious damage to the cellular morphology, and the cells shrank into granules with partial cell necrosis.

### Differential Expression of Antiviral Genes Following PEDV Infection in IPEC-J2 Cells

**Figure 2:**
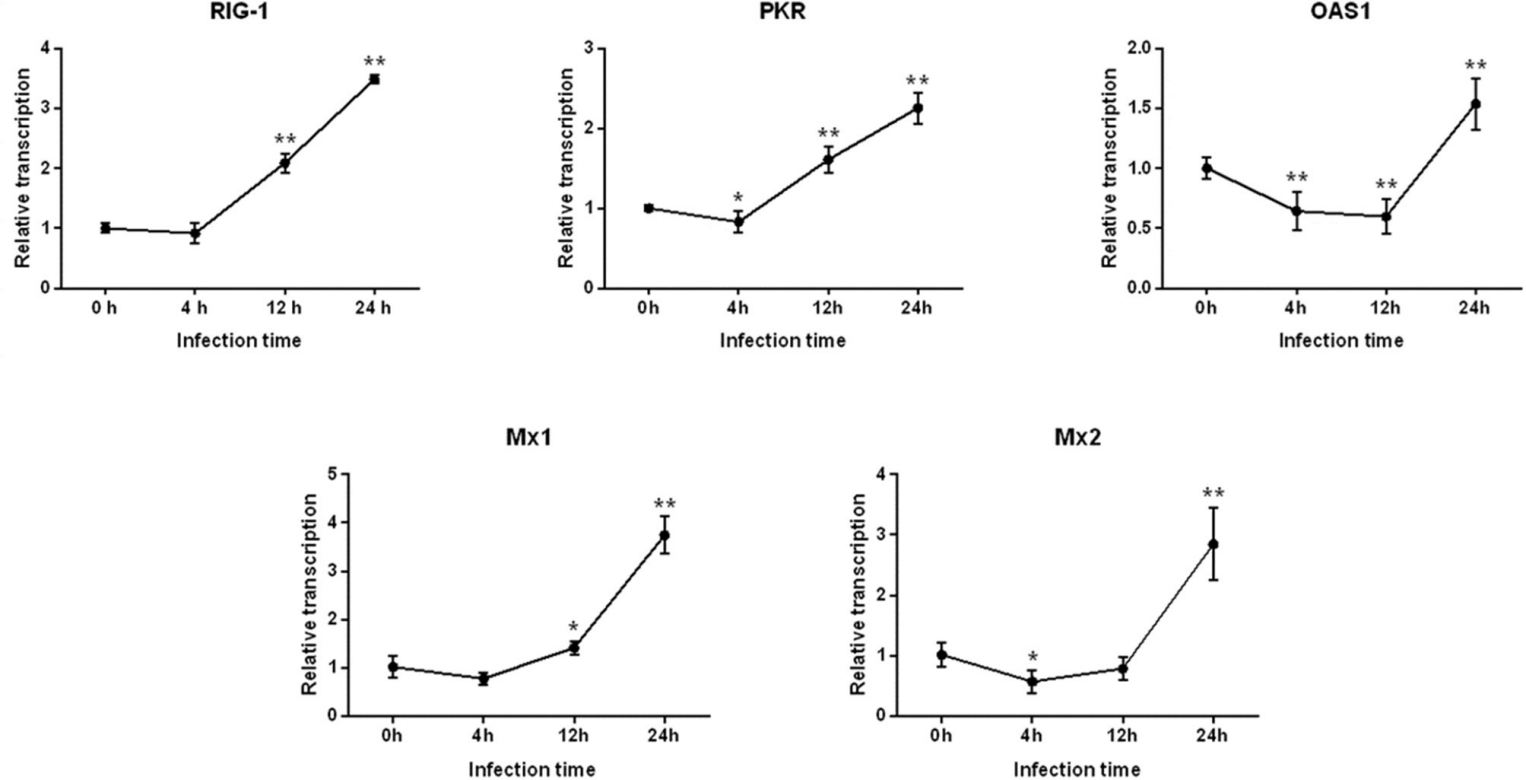
Changes in the level of *RIG-1, PKR, OAS1, Mx1*, and *Mx2* gene expression after PEDV infection of IPEC-J2 cells. *Represents a significant difference relative to the control group (0 h) (*P* < 0.05), and **Represents an extremely significant difference relative to the control group (*P* < 0.01).

As shown in Figure 2, after 4 h of PEDV infection, the expressions of all antiviral genes decreased to various degrees, for which the level of *PKR* and *Mx2* gene expressions decreased significantly (*P* < 0.05), and expression of the *OAS1* gene was extremely significantly different (*P* < 0.01). Subsequently, the mRNA levels of *RIG-1, PKR*, and *Mx1* gene began to increase at 12 h post-infection, whereas the expression of *OAS1* continued to significantly decline (*P* < 0.01). Finally, the expressions of all antiviral genes were highly significantly up-regulated (*P* < 0.01), and peaked at 24 h.

### Correlation Analysis of the Differences in the Level of Antiviral Gene Expression and Prediction of Protein-Protein Interactions Following PEDV Infection

**Figure 3:**
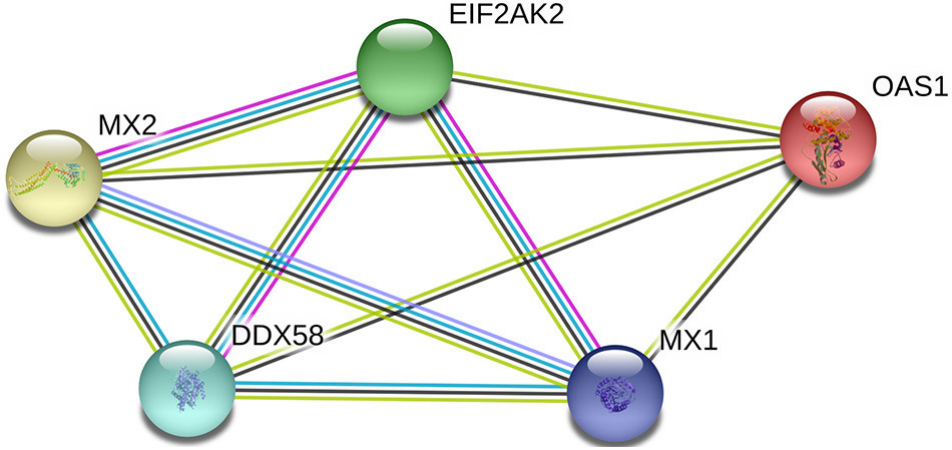
Network diagram of proteins with porcine RIG-1 (also known as DDX58), PKR (also known as EIF2AK2), OAS1, Mx1, and Mx2 proteins.

The inter-relationship between the expression of *RIG-1, PKR, OAS1, Mx1, and Mx2* were analyzed by String 11.0, and presented in an interaction network (Figure 3). Therefore, we analyzed the correlation between the transcription levels of these five genes. The results were presented in Table 1. The transcriptions of five antiviral genes were found to be significantly and positively correlated with each other (*P* < 0.01).

**Table 1:** The correlation matrix among expression levels of antiviral genes.

**Genes**	***RIG-1***	***PKR***	***OAS1***	***Mx1***	***Mx2***
*RIG-1*	1.000				
*PKR*	0.973^**^	1.000			
*OAS1*	0.671^**^	0.623^**^	1.000		
*Mx1*	0.940^**^	0.912^**^	0.776^**^	1.000	
*Mx2*	0.847^**^	0.800^**^	0.798^**^	0.947^**^	1.000

^*^Means significant correlation (P < 0.05),

** *Means extremely significant correlation (P < 0.01)*.

### Differences in the Level of Inflammatory Factor Transcription After PEDV Infection in IPEC-J2 Cells

**Figure 4:**
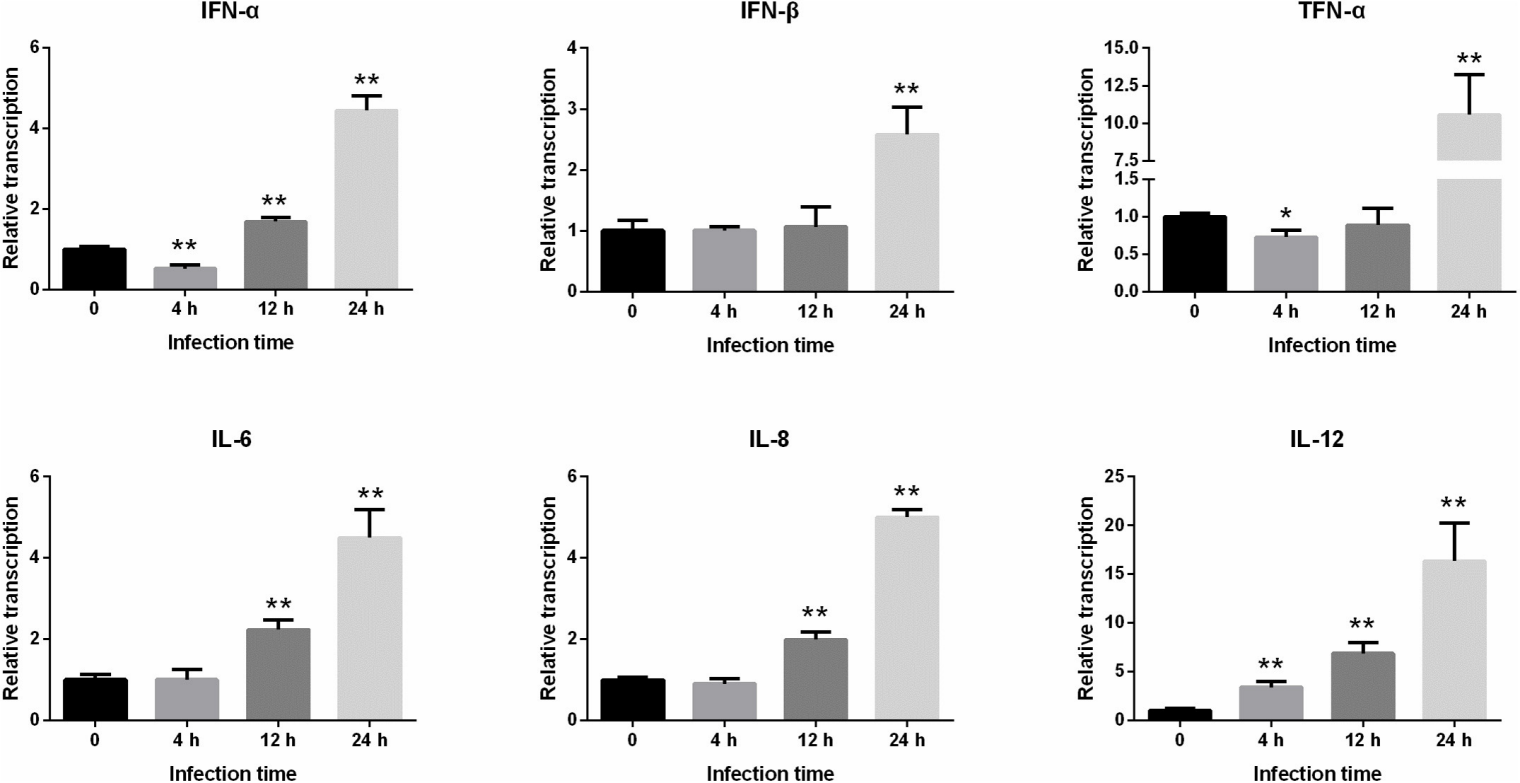
Changes in the level of *IFN-*α*, IFN-*β*, TNF-*α*, IL-6, IL-8*, and *IL-12* gene expression after PEDV infection of IPEC-J2 cells. *Represents a significant difference relative to the control group (0 h) (*P* < 0.05), and **Represents an extremely significant difference relative to the control group (*P* < 0.01).

We next examined the differences in the levels of transcriptions of some important immune factors in IPEC-J2 cells within 24 h following PEDV infection (Figure 4). It was found that the expressions of *IFN-*α and *TNF-*α significantly decreased 4 h after infection, but *IL-12* gene increased significantly (*P* < 0.01). After infection for 12 h to 24 h, the expressions of *IFN-*α, *IFN-*β, *TNF-*α, *IL-6, IL-8*, and *IL-12* began to increase continuously, and the levels of transcriptions of these inflammatory factors peaked at 24 h.

## Discussion

Recently, the relationship between PEDV infection and host natural immunity has become an issue of keen interest throughout the world. A systematic understanding of the interaction between PEDV and innate immunity can provide us with new strategies for the preventive treatment of PED. PEDV is positive sense single-stranded RNA virus coated with a capsid for which the pathogenesis is associated with the binding of the S1 subunit region of the virus-encoded S protein to the receptor gene (APN) expressed on porcine small intestinal epithelial cells (15). Then PEDV enters the host cell via membrane fusion, and begins to replicate and proliferate in the host cell, producing double-strained RNA (dsRNA) (16). Studies have shown that RIG-1 is a receptor that recognizes viral dsRNA in the cytoplasm (17). Moreover, RIG-1-like receptors (RLRs) bind to viral dsRNA by recognizing pathogen-related molecular patterns. In addition, the N-terminal CARD structure of RLRs can interact with the downstream adaptor protein, IPS-1, to activate the downstream TANK-binding kinases, TBK1 and IKKε, activate the transcription factor, NF-κB, and IFN regulatory factor 3 (IRF-3) to induce type 1 IFN production (10, 11). At present, substantial progress has been made in the study of PEDV-mediated antagonization of the natural innate immune response. There are two main viral escape mechanisms: (1) blocking the RIG-1/IPS1 signaling pathway by hiding its dsRNA; and (2) encoding an IFN antagonist protein to attack the transmission of molecular signals in antiviral signaling pathways (18–20).

In the present study, we found that although no significant differences were observed, the expression of *RIG-1* began to decrease within 4 h after PEDV infection in IPEC-J2 cells. In addition, IFN-α transcribed by cells was also substantially reduced at this time point, which may be the result of PEDV antagonizing the host innate immune response, suggesting that the virus began to invade the small intestinal cells 0–4 h after challenge. Our study showed that PEDV infected IPEC-J2 cells by interfering with the RIG-1-mediated signaling pathway to inhibit *IFN-*α transcription, thereby perpetuating an extended infection in the host. Subsequently, the level of *RIG-1, IFN-*α, and *IFN-*β gene expression began to increase at 12–24 h post-infection, and obvious lesions were observed simultaneously, indicating that host innate immunity plays an important role in immune regulation at this time point.

When IFN-I (IFN-α/β) is secreted extracellularly through various methods, it binds to IFNAR1/2 and activates the Janus kinases/Signal transducers and activators of transcription (Jak/Stat) signaling pathway, which in turn induces transcriptional activation of ISGs. ISGs are effector molecules that mediate cellular innate immune responses to viral infections, including PKR, OAS1, Mx1, and Mx2. PKR is a serine/threonine protein kinase that can be activated by dsRNA produced by viral replication, which can inactive the eukaryotic translation initiation factor, eIF-2α, resulting in blocking the entry of viral or host mRNA into the ribosome to form the initiation complex (21). OAS1 is an interferon-induced antiviral protease that can recognize dsRNA and activate endonuclease (RNaseL), to degrade both viral mRNA and cellular RNA (22). Studies have shown that porcine OAS1 can effectively inhibit the infectious activity of Japanese encephalitis virus, pseudorabies virus, and porcine respiratory and reproductive syndrome virus (23). Mx protein is induced by IFN-I and IFN-II and is associated with intracellular vesicle transportation and intracellular homeostasis. Moreover, it exhibits extensive anti-RNA virus activity. Sun et al. successfully constructed an Mx1 or Mx2 over-expressing monkey kidney epithelial cell line, MARC-145, and found that both could effectively inhibit the replication of PEDV, albeit Mx2 displays a stronger anti-PEDV activity compared to Mx1 (24). Our study found that the levels of *PKR, OAS1*, and *Mx2* expression decreased at 0–4 h after the attack, indicating that PEDV may interfere with antiviral gene expression and inhibit the cellular innate immune response. The expression of *PKR, OAS1, Mx1*, and *Mx2* peaked at 24 h. In addition, in IPEC-J2 cells infected with PEDV, the expressions of the *RIG-1, PKR, OAS1, Mx1*, and *Mx2* genes were significantly and positively correlated to each other, likely because these genes are induced through the Jak/Stat signaling pathway, and their expression profiles are simulations similar in response to invasion of the same virus (25). Thus, our findings suggested that these five antiviral genes interacted to regulate PEDV infection.

Inflammation mediated by the innate immune system is also an important indicator by which host disease resistance can be measured. Therefore, research based on proinflammatory cytokines is helpful for clarifying the pathogenesis of diseases and the associated host immune mechanisms against infection (26). IFN-α and IFN-β have antiviral, antitumor, antiparasitic, and immune regulatory effects. TNF-α, IL-6, IL-8, and IL-12 are important cytokines involved in the immune response and mediating the inflammatory response (13). In this study, the levels of proinflammatory cytokine transcriptions were monitored and the cytopathic conditions were also observed to explore the immune status of the cells following viral infection. The results showed that no obvious lesions were observed in the cells at 4 h after infection, and there was no significant difference in the expressions of other inflammatory factors, except *IL-12* at this time point. The expressions of *IFN-*α and *TNF-*α were also inhibited, indicating that the cellular inflammatory reaction was minor at this time point. At 12 h post-infection, the cells began to shrink and the lesions were evident. At 24 h, the cellular pathological changes were the most severe. In addition, the levels of inflammatory factor transcriptions increased continuously from 12 to 24 h after infection, and the expression levels peaked at 24 h, indicating that the inflammatory reaction of the cells was the most intense at this time point.

Cao et al. reported that no pathological changes were observed in IPEC-J2 cells with PEDV infection for 72 h, and the expression of *RIG-1* and *IFN-*β was inhibited continuously (27). In the present study, we found that the expression of antiviral genes (e.g., *PKR, OAS1*, and *Mx2*) and inflammatory cytokines (e.g., *IFN-*α and *TNF-*α) were significantly reduced within 0–4 h post-infection. However, all antiviral genes and inflammatory cytokines were up-regulated from 12 to 24 h, and cytopathic changes were observed during this period. These results suggested that the expressions of the antiviral genes and inflammatory cytokines are positively correlated with the severity of the cytopathic effect following PEDV infection of IPEC-J2 cells. Activation of these genes might be a hallmark of PEDV infection.

In this study, we preliminarily investigated the expression patterns of the antiviral genes *RIG, PKR, OAS1, Mx1, and Mx2*, which are involved in the host innate immune mechanisms during PEDV infection of IPEC-J2 cells. We then analyzed the inflammatory reaction mediated by PEDV infection. Our future research will involve constructing an antiviral gene overexpressing or knock-out IPEC-J2 cell line, and evaluating its resistance to PEDV. Such findings will provide a more direct theoretical basis for the development of genetic breeding strategies against swine epidemic diarrhea in the future.

## Data Availability Statement

All datasets generated for this study are included in the article/Supplementary Material.

## Author Contributions

WB and SWu conceived and supervised the study. SWa and HW designed the experiments. SWa, JW, and FW performed the experiments. SWa and ZW analyzed the data. SWa and JW contributed to the writing of the manuscript.

### Conflict of Interest

The authors declare that the research was conducted in the absence of any commercial or financial relationships that could be construed as a potential conflict of interest.
