# Supplementary material for: Expression Pattern Analysis of Antiviral Genes and Inflammatory Cytokines in PEDV-Infected Porcine Intestinal Epithelial Cells
Source: Front Vet Sci. 2020 Feb 18;7:75. doi: 10.3389/fvets.2020.00075 (PMC7040077; doi:10.3389/fvets.2020.00075)
Supplement: Supplementary file 1 [file Data_Sheet_1.PDF]

Expression Pattern Analysis of Antiviral Genes and Inflammatory Cytokines in PEDV-infected Porcine Intestinal Epithelial Cells

Shiqin Wang[1†], Jiayun Wu[1†], Fang Wang[1], Haifei Wang[1,2], Zhengchang Wu[1,2], Shenglong Wu[1,2], Wenbin Bao[1,2*]

Supplementary materials

Table S1. Information of qRT-PCR Primers

Gene	Primer Sequence (5 '→ 3')	GenBank Accession number	Product Length
RIG-1	F: AAGAAGAGTACCACTTAAACCCAG<br>R: ATGCCTTCATCTGCCACCGA	NM213804.2	256 bp
PKR	F: TTGCGAGAAGGTAGAGCGTG<br>R: TCATTCCCATCCCAGCAACC	XM021085862.1	90 bp
OAS1	F: CCAACAGGTTCAGACAGCCT<br>R: GAGGAGCCACCCTTCACAAC	NM214303.2	245 bp
Mx1	F: GTCATCGGGGACCAGAGTTC<br>R: TCCCGGTAACTGACTTTGCC	NM214061.2	164 bp
Mx2	F: CCAGAGGCAGCGGAATCAT<br>R: TTTGCGTATTTCCCGCTCCA	NM001097416.1	143 bp
IFN-α	F: TTCTGCACTGGACTGGATC<br>R: TCTGTGGAAGTATTTCCTCACAG	KF414740.1	103 bp
IFN-β	F: GCTAACAAGTGCATCCTCCAAA<br>R: CCAGGAGCTTCTGACATGCCA	JN391525.1	124 bp
TNF-α	F: CCTACTGCACTTCGAGGTTATC<br>R: GCATACCCACTCTGCCATT	JF831365.1	158 bp
IL-6	F: CTCTGTCTTAGGGCGTCC<br>R: CAAGGAGGTACTGGCAGAAA	JQ839263.1	164 bp
IL-8	F: CCACACCTTTCCACCCCAAA<br>R: TTGTTGCTTCTCAGTTCTCTTCA	NM213867.1	179 bp
IL-12	F: CAGGCCCAGGAATGTTCAAA<br>R: CGTGGCTAGTTCAAGTGGTAAG	NM213993.1	166 bp
GAPDH	F: ACATCATCCCTGCTTCTACTGG<br>R: CTCGGACGCCTGCTTCAC	AF017079.1	188 bp

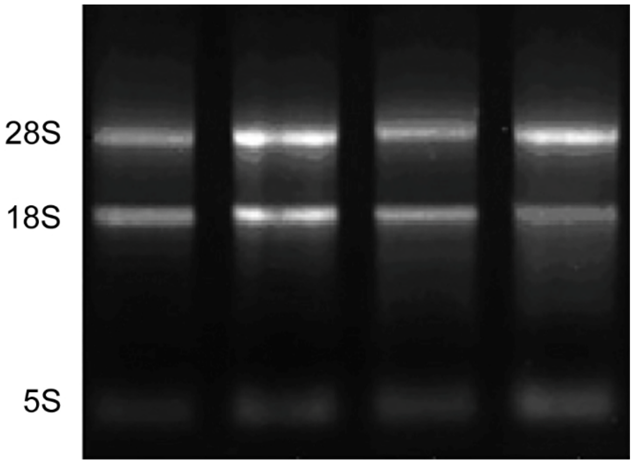

Fig. S1 Denaturing agarose electrophoresis results of cell total RNA

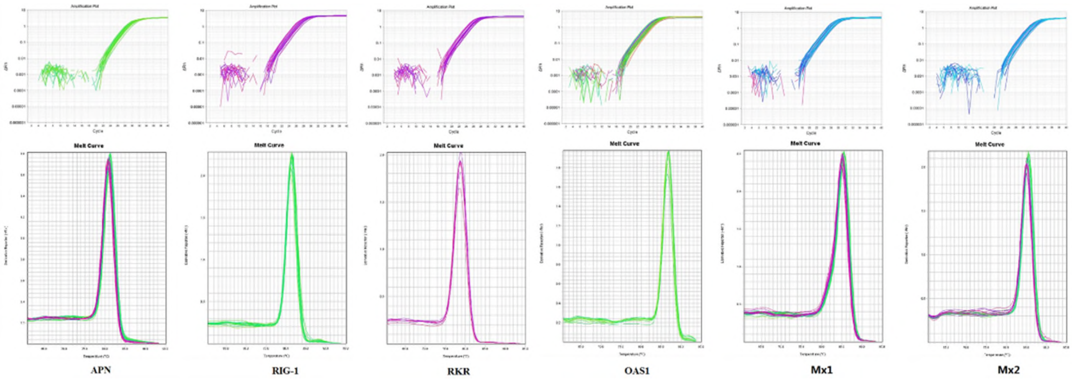

Fig. S2A Amplification curve and melting curve of *RIG-1*, *PKR*, OAS1, *Mx1* and *Mx2* genes

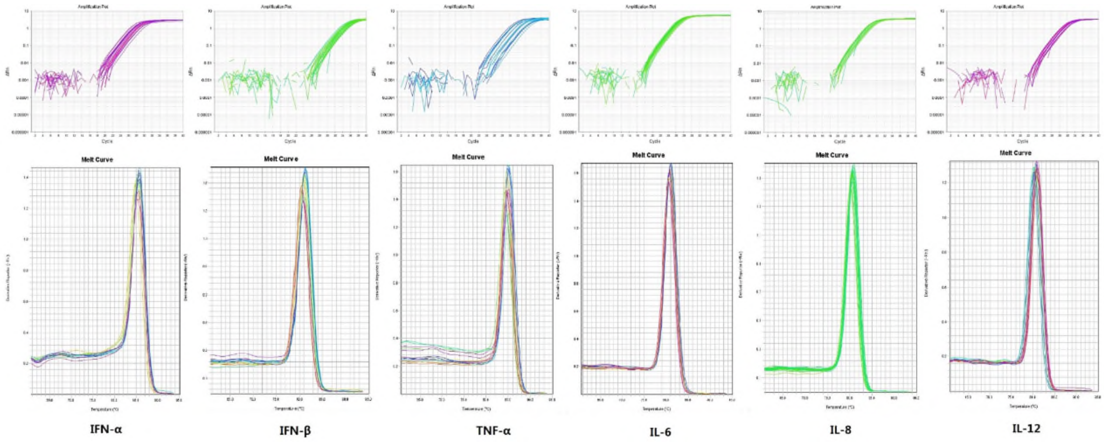

Fig. S2B Amplification curve and melting curve of *IFN-α*, *IFN -β*, *TNF-α*, *IL-6*, *IL-8* and *IL-12* genes
